# An Unusual Case of Systemic Inflammatory Myofibroblastic Tumor with Successful Treatment with ALK-Inhibitor

**DOI:** 10.1155/2014/470340

**Published:** 2014-06-18

**Authors:** Sanjivini V. Jacob, John D. Reith, Angerika Y. Kojima, William D. Williams, Chen Liu, Lizette Vila Duckworth

**Affiliations:** ^1^Department of Pathology, Immunology and Laboratory Medicine University of Florida College of Medicine, University of Florida College of Medicine, P.O. Box 100275, 1600 SW Archer Road, Gainesville, FL 32610-0275, USA; ^2^Lake Erie Osteopathic College of Medicine, Bradenton, FL 34211, USA; ^3^Department of Pathology, North Broward Health, Pompano Beach, FL 33064, USA

## Abstract

Systemic inflammatory myofibroblastic tumor is an exceedingly rare entity. A 45-year-old Hispanic female presented with a 6-month history of left-sided thigh pain, low back pain, and generalized weakness. PET/CT scan revealed abnormal activity in the liver, adrenal gland, and pancreas. MRI of the abdomen demonstrated two 6-7 cm masses in the liver. MRI of the lumbar spine demonstrated lesions in the L2 to L4 spinous processes, paraspinal muscles, and subcutaneous tissues, as well as an 8 mm enhancing intradural lesion at T11, all thought to be metastatic disease. A biopsy of the liver showed portal tract expansion by a spindle cell proliferation rich in inflammation. Tumor cells showed immunoreactivity for smooth muscle actin and anaplastic lymphoma kinase 1 (ALK1). Tissue from the L5 vertebra showed a process histologically identical to that seen in the liver. FISH analysis of these lesions demonstrated an ALK (2p23) gene rearrangement. The patient was successfully treated with an ALK-inhibitor, Crizotinib, and is now in complete remission. We present the first reported case, to our knowledge, of inflammatory myofibroblastic tumor with systemic manifestations and ALK translocation. This case is a prime example of how personalized medicine has vastly improved patient care through the use of molecular-targeted therapy.

## 1. Introduction

Inflammatory myofibroblastic tumor (IMT) is a mesenchymal neoplasm more commonly occurring during the first two decades of life. IMTs can arise in various anatomic locations [1–6] but occur primarily in the lung [7–9], orbit, retroperitoneum, or abdominopelvic region [10–19]. Lesions may be multifocal. IMT is characterized by a proliferation of myofibroblasts admixed with an inflammatory component of lymphocytes, eosinophils, and plasma cells. Approximately half of IMTs have a rearrangement of the anaplastic lymphoma kinase (ALK) locus on chromosome 2p23, leading to aberrant ALK expression [20, 21]. ALK rearrangements are associated with younger presentation and a more indolent behavior [22]. Local recurrence may occur after surgery with a low risk of distant metastases. A small fraction of IMTs behave more aggressively. The management of IMT can be challenging as there are no established medical treatment protocols [1]. We present the first reported case of systemic IMT with ALK gene rearrangement in a 45-year-old female that responded dramatically to an ALK-inhibitor. This case suggests a therapeutic breakthrough for ALK-positive IMTs using an ALK inhibitor.

## 2. Case Report

A 45-year-old obese Hispanic female, status post cholecystectomy, presented initially with left leg pain, low back pain, generalized weakness, and right-sided blurred vision. Physical examination at that time revealed an abnormal left leg gait and a right lateral strabismus. Laboratory studies revealed mildly elevated liver enzymes (AST 87, ALT 129, total bilirubin 0.9, and alkaline phosphatase 88). Imaging revealed multiple small liver masses and lesions in the lumbar spine. A liver biopsy was performed and was initially interpreted as nonspecific subacute and chronic inflammation with abscess formation. No granulomata, organisms, or evidence of malignancy were seen, and the patient was treated with intravenous antibiotics. Due to high suspicion of tumor in the lumbar region, an exploration of the L3-L4 region was also performed. Operatively, the nerve roots were found to be inflamed in the area, and a proteinaceous cyst was also seen. The patient was subsequently discharged after extensive workup and cultures proved negative.

**Figure 1:**
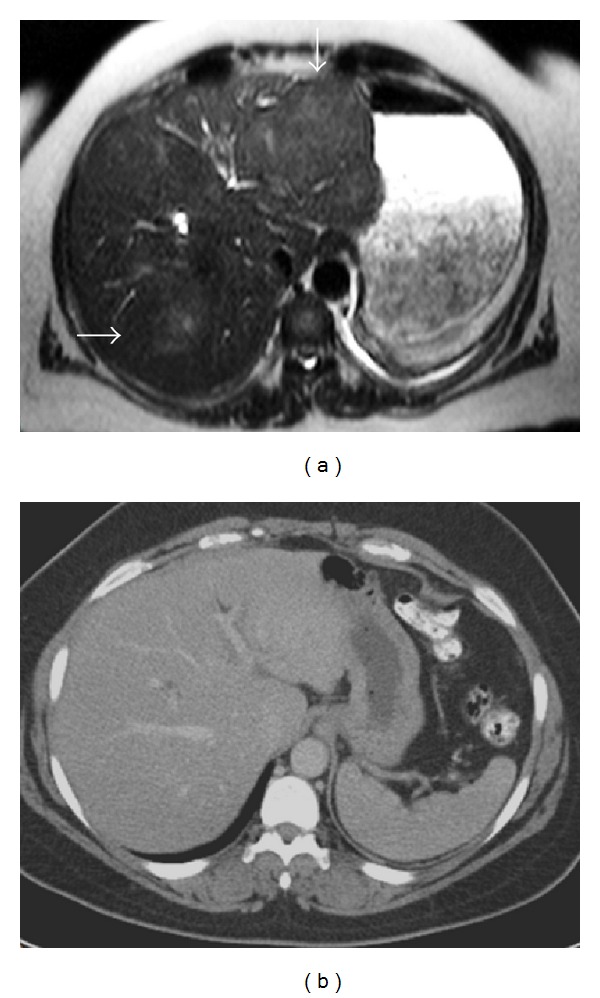
Radiologic appearance of IMT lesions in the liver before (a) and after treatment (b) with ALK-inhibitor. (a) MRI of the abdomen with and without Gadolinium before treatment demonstrates vague poorly defined liver masses in the right and left lobes (arrows) each measuring approximately 6-7 cm. (b) CT of the abdomen obtained at 15-month follow-up demonstrates resolution of the ill-defined liver masses bilaterally following treatment with ALK-inhibitor.

The patient was readmitted three months after initial discharge due to worsening of low back pain, pain on ambulation, and bilateral lower extremity weakness and numbness, as well as an increase in size of the hepatic and spinal lesions on imaging. Physical exam now showed decreased pinprick, dysesthesias, and weakness with dorsiflexion in the lower extremities bilaterally. Right third cranial nerve palsy was also noted. Repeat MRI of the lumbar spine with and without Gadolinium revealed diffuse enhancing bony lesions involving the L2 to L4 spinous processes, posterior paraspinal muscles, and posterior subcutaneous tissues, and an 8 mm enhancing intradural lesion at T11, all thought to be metastatic disease. MRI of the abdomen with and without Gadolinium demonstrated a poorly defined 6-7 cm mass in the left lobe of the liver and a 6 cm mass in the right lobe of the liver (Figure 1(a)). PET/CT demonstrated abnormal activity in the liver and focal increased activity in the left adrenal gland and head of the pancreas. CA125 was elevated at 88 U/mL (normal range, less than 35 U/mL) but CEA, CA15-3, AFP, and CA19-9 were all within normal range. Serum protein electrophoresis was also performed and was negative for monoclonal gammopathy. The lumbar area was reexplored at that time. Bone and ligamentous material were sent to pathology. A repeat liver biopsy was also performed.

## 3. Pathologic Findings

**Figure 2:**
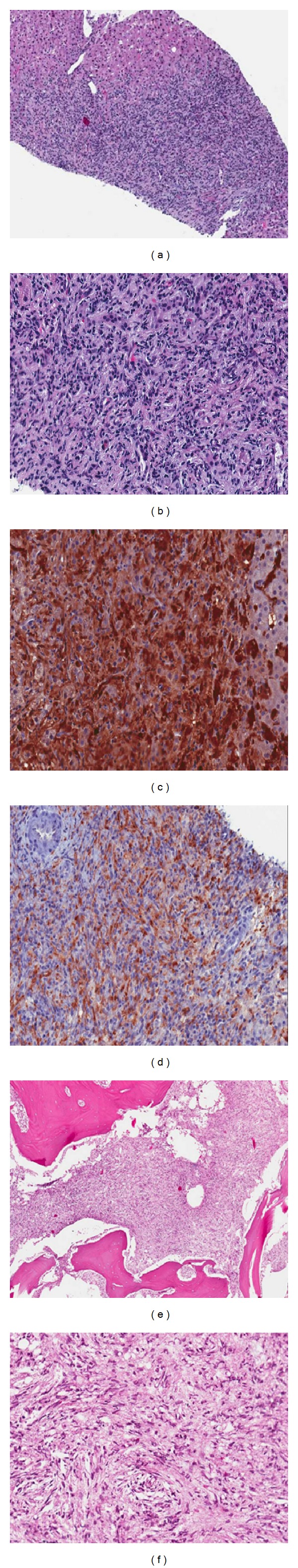
Histologic appearance of IMT lesions in liver and lumbar spine. (a) and (b) Liver tissue with marked inflammatory cells and spindle cell proliferation expanding portal areas and extending into parenchyma. Adjacent normal liver is seen on upper left corner of (a), (H&E), 5x (a) and 20x (b). (c) Spindle cells show strong and diffuse immunoreactivity to SMA, 20x. (d) Spindle cells are immunoreactive for cytoplasmic anaplastic lymphoma kinase protein (ALK-1), 20x. (e) and (f) Lumbar spine is infiltrated by similar mixture of inflammatory and spindled cells expanding the bone marrow, H&E, 5x and 20x.

**Figure 3:**
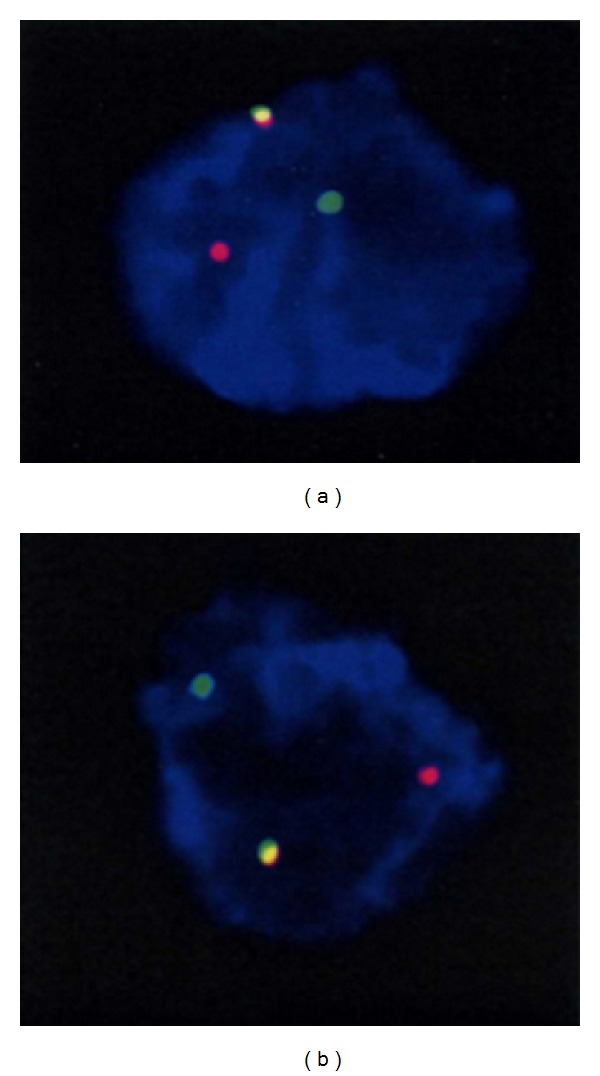
FISH analysis using dual color ALK break apart probe of the liver (a) and lumbar lesions (b) showing abnormal ALK (2p23) gene arrangement (translocation). (a) and (b) This is a “break-apart” probe system, where the fused red/green (overlap as yellow) represents the normal ALK gene locus and the separated red and green signals represent an underlying ALK rearrangement (i.e., translocation). Interphase FISH analysis of at least 200 interphase nuclei was performed with a dual color ALK break probe apart set to detect rearrangement of the ALK locus that is commonly associated with anaplastic large cell lymphoma. The break-apart signal pattern (1R1G1F, one red, one green, and one fusion signal) was observed in 11.5% of the analyzed nuclei in the liver (a) and in 12.5% of the analyzed nuclei in the lumbar spine (b). The normal reference is 1.7%, and as such, this represents an* abnormal* result indicative of ALK gene rearrangement with an unknown chromosomal locus.

Histologic examination of the liver and spine lesions revealed a spindle cell proliferation admixed with an inflammatory infiltrate composed of lymphocytes, plasma cells, histiocytes, and eosinophils (Figure 2). The spindle cells were bland in appearance, and no necrosis or atypical mitoses were seen. Immunohistochemical studies revealed that the spindle cells were diffusely immunoreactive for antibodies to smooth muscle actin (SMA), CD68, and exhibited cytoplasmic staining with ALK-1 but were negative for CD117, CD1a, CD23, CD21 (Langerhan cell and dendritic cell markers), EBV, LCA (hematopoetic markers), HepPar1 (hepatocellular marker), CD34, and S-100 protein. A Giemsa stain was also negative. Subsequently, FISH analysis of both the liver and bone lesions showed an ALK (2p23) gene rearrangement (Figure 3). The patient was given a diagnosis of ALK-rearranged systemic inflammatory myofibroblastic tumor. She subsequently was treated with the ALK-inhibitor Crizotinib and is currently in complete remission (Figure 1(b)) after 27-month follow-up.

## 4. Discussion

Inflammatory myofibroblastic tumor is defined by the World Health Organization as a distinctive lesion of myofibroblastic spindle cells with an inflammatory infiltrate composed of lymphocytes, eosinophils, and plasma cells [23]. It is a rare entity occurring mostly in children and young adults with a mean age of 10 years at diagnosis. Most cases occur within the first 2 decades of life, with a slight female preponderance. These tumors are more commonly found in the soft tissue and viscera of the lungs, mesentery, and abdominopelvic region but can involve any organ system [1–19]. The mainstay of treatment for this tumor is complete surgical excision, although local recurrence and spread have been reported, especially when complete resection is not achieved [1, 10, 13, 15, 19, 22, 24, 25]. Several cases have been treated with chemotherapy, radiation, and/or corticosteroids [1, 3, 10, 19, 24]. Systemic or malignant transformation of IMT is rare and has only been reported in a few cases [19, 22, 25].

Pathologic features of IMTs including tumor size, mitotic rate, presence or absence of necrosis, and cellular atypia do not appear to correlate well with clinical outcome. A small subset of IMTs demonstrate histologic progression to malignancy with increased cellularity/pleomorphism of spindled, round, or polygonal cells with vesicular nuclei, high mitotic rate, and atypical mitoses [26]. However, cases lacking these histologic features have been found to behave in an aggressive manner, and conversely cases with worrisome histologic features have been found to behave indolently [22]. In recent years, a highly aggressive intra-abdominal variant of IMT has been described with epithelioid-to-round cell morphology with nuclear membrane or perinuclear ALK staining and predilection for male patients, which has been termed “epithelioid inflammatory myofibroblastic sarcoma” to convey the malignant nature of this tumor and close relation to IMT [27].

While the nature of IMT is debated and historically thought to be driven by an inflammatory process [22, 27], Griffin et al. demonstrated a chromosomal aberration associated with these tumors [21]. In their study, they found that half of IMTs contained a breakage in band p22–24 of chromosome 2, with specific involvement of 2p23 leading to ALK overexpression [21]. ALK overexpression is typically detectable by immunohistochemistry, which demonstrates cytoplasmic reactivity in IMT, and is also detectable by FISH. Fusion partners of ALK in IMT include TPM3 and TPM4 [28, 29], CARS [28, 30], clathrin [31], RAN-BP2 [32], ATIC [33], SEC31L1 [34], and PPF1BP1 [8]. ALK rearrangement has been associated with younger age and local recurrence, but not with distant metastasis [22]. In a study by Coffin et al., 59 cases of IMT were studied, of which 6 were found to have distant metastasis [22]. All six cases with distant metastasis were reported to be ALK negative. To our knowledge, our case represents the first reported case of ALK-translocated systemic IMT in a patient presenting with synchronous tumors in the liver and lumbar spine.

Based on a recent study in the New England Journal of Medicine of successful use of ALK-inhibitor in a treating a patient with ALK-positive IMT [24], our patient was treated with the ALK inhibitor Crizotinib with successful resolution of her lesions and symptoms. After 27-month follow-up, the patient remained in complete clinical and radiologic remission.

In summary, this case represents a distinct presentation of inflammatory myofibroblastic tumor with systemic involvement and ALK gene rearrangement. This case illustrates how personalized medicine through the use of molecular testing has made individual targeted therapy successful.
